# Supplementary material for: Optimization of Liposomes for Antigen Targeting to Splenic CD169+ Macrophages
Source: Pharmaceutics. 2020 Nov 25;12(12):1138. doi: 10.3390/pharmaceutics12121138 (PMC7760819; doi:10.3390/pharmaceutics12121138)
Supplement: Supplementary file 1 [file pharmaceutics-12-01138-s001.pdf]

pharmaceutics

MDPI

Supplementary Materials: Optimization of Liposomes for Antigen Targeting to Splenic CD169^{+} Macrophages

Maarten K. Nijen Twilhaar, Lucas Czentner, Joanna Grabowska, Alsya J. Affandi, Chun Yin Jerry Lau, Katarzyna Olesek, Hakan Kalay, Cornelus F. van Nostrum, Yvette van Kooyk, Gert Storm and Joke M.M. den Haan

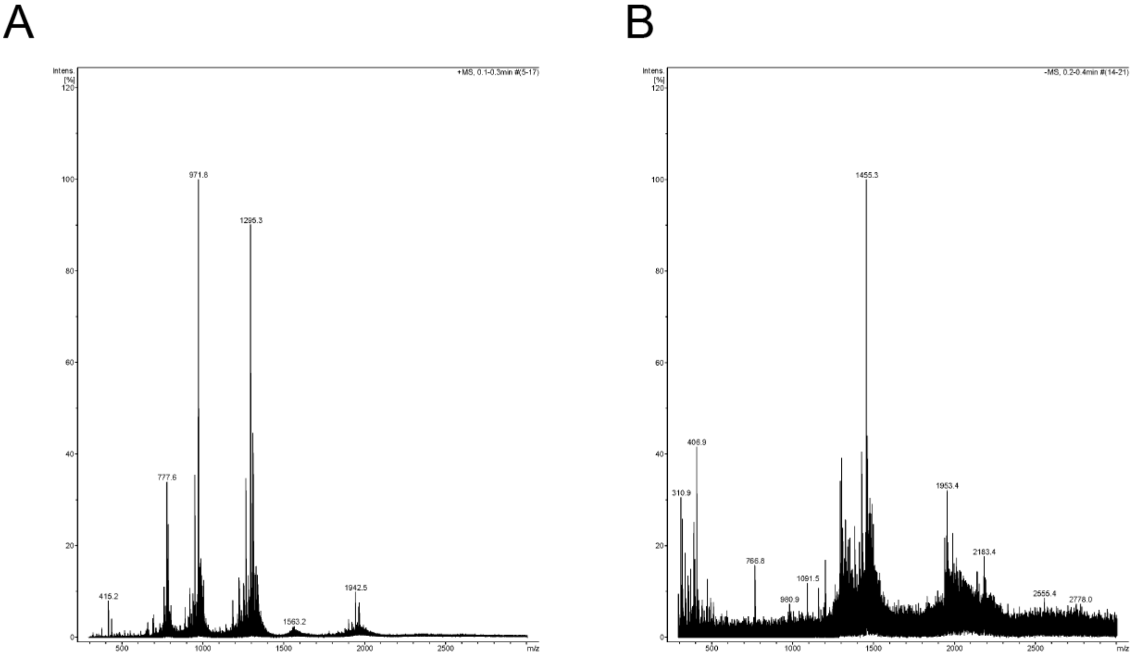

Figure S1. Mass spectra of synthesized OVA-peptides. (**A**) $OVA_{247-279}$ peptide was analyzed in positive mode, fragment ions $[M+2H]^{2+}$ = 1942.5 $[M+3H]^{3+}$ = 1295.3; $[M+4H]^{4+}$ = 971.8 and $[M+5H]^{5+}$ = 777.6 confirmed peptide identity. (**B**) 5-FAM-Lys-$OVA_{247-279}$ peptide was analyzed in negative mode, fragment ions $[M-2H]^{2-}$ = 2183.4; $[M-3H]^{3-}$ = 1455.3 and $[M-4H]^{4-}$ = 1091.5 confirmed peptide identity.

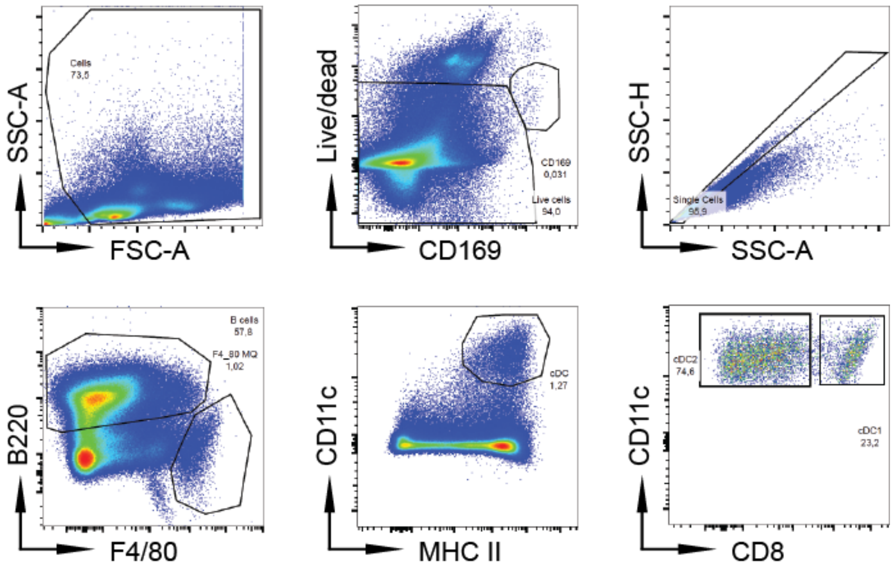

Figure S2. Gating strategy for splenic B cells, macrophages and DC populations. After gating out debris, live CD169+ MΦ were identified based on their CD169 expression and autofluorescence. Remaining live cells were further gated based on B220 for B cells and F4/80 for red pulp MΦ, abbreviated as RP MΦ. Classical DCs were identified based on their CD11c and MHC class II expression and further subdivided in CD8 expressing cDC1 and non CD8 expressing cDC2.

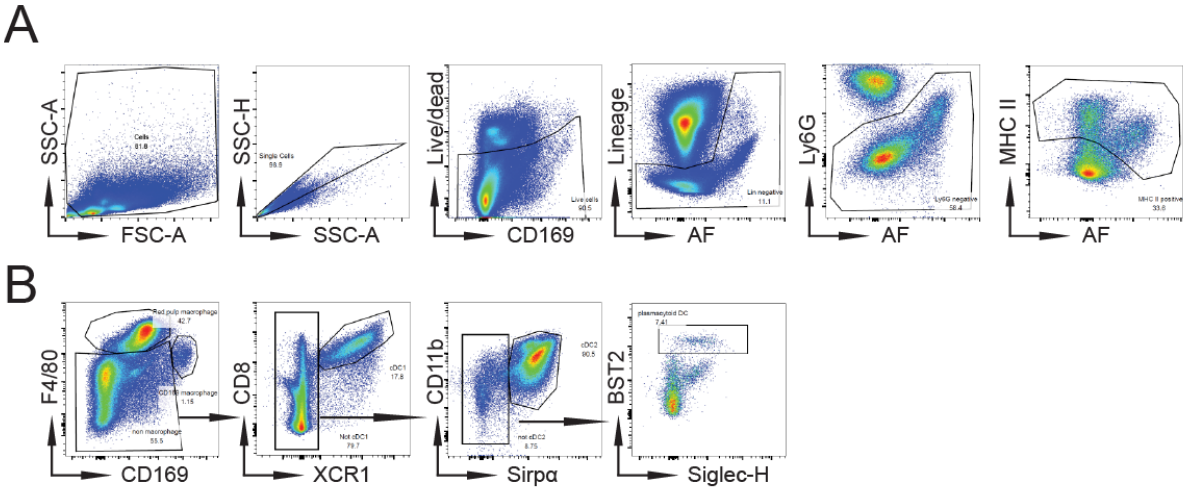

Figure S3. Gating strategy for macrophage and DC populations. (A) After gating out debris, single and live cells were gated. Subsequently, lineage cells (CD3+ T cells, CD19+ B cells and CD56+ NK cells) and Ly6G+ granulocytes were excluded, after which MHCII and auto fluorescent (AF) were selected for tSNE analysis. (B) Cells were gated as in a, subsequently red pulp MΦ and CD169+ MΦ were identified based on their high F4/80 or high CD169 expression, respectively. Subsequently, cDC1 and cDC2 were identified based on their CD8/XCR1 or CD11b/Sirpα expression, respectively. Next, pDC were identified based on their BST2 expression.

pharmaceutics

MDPI

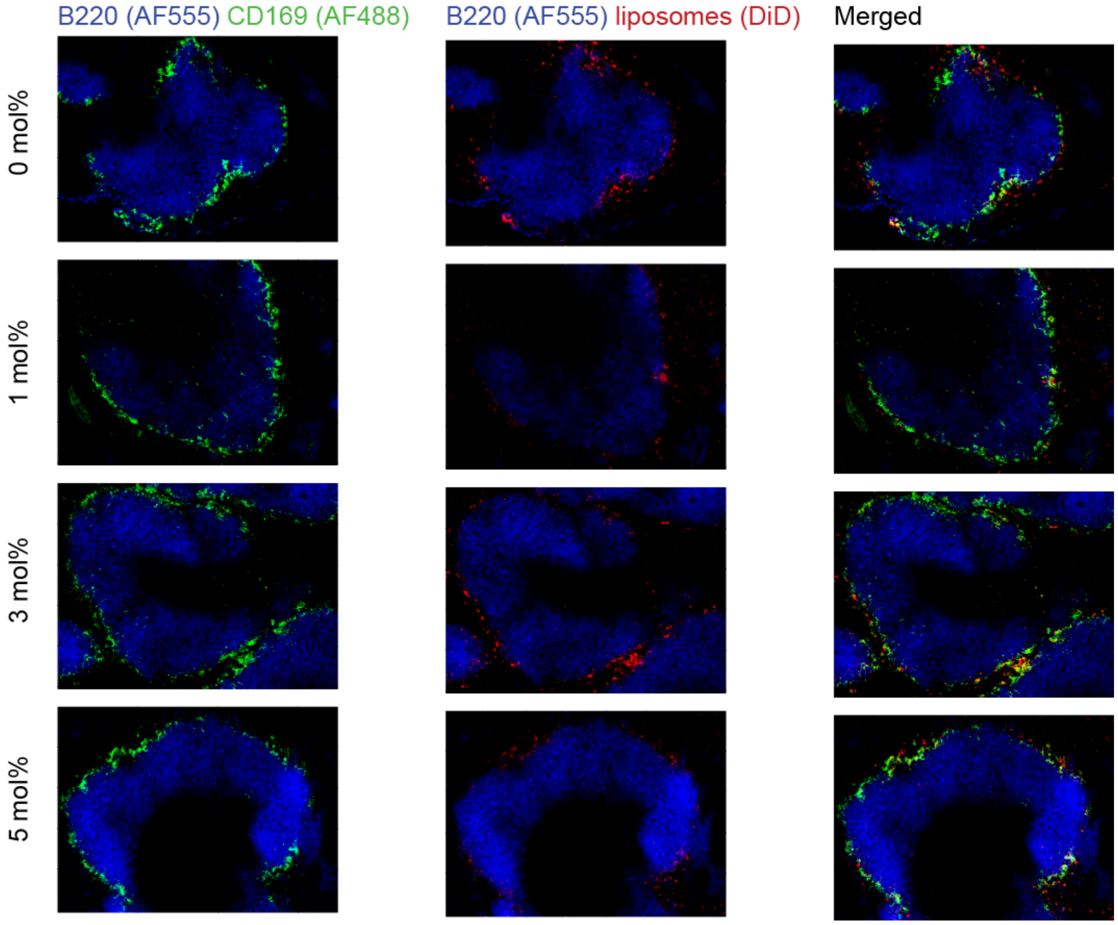

Figure S4. Intravenously injected liposomes accumulate in close proximity to CD169⁺ MΦ. DiD-containing liposomes (22.5 nmol phospholipid), supplemented with adjuvant, were IV injected in mice. After 2h spleens were isolated for immunofluorescence microscopy analysis. Indicated is the liposomal DiD immunofluoresence in red, B cells were stained with anti-B220 in blue and the CD169 staining in green.

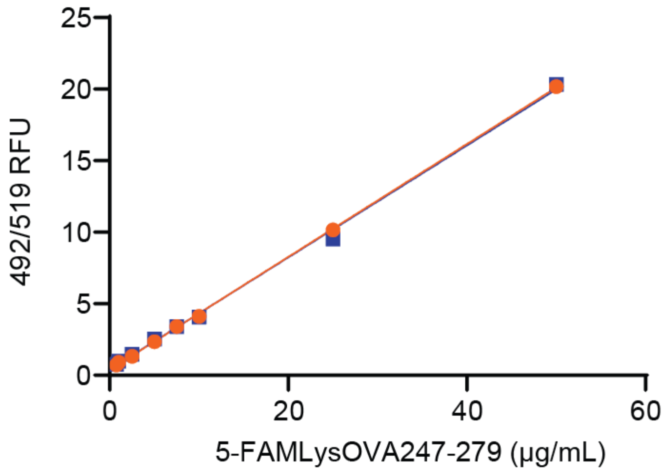

Figure S5. Linear range detection of fluorescent OVA-peptide. The relatively fluorescent unit (RFU) of 0.75 µg/mL to 50 µg/mL fluorescent OVA-peptide spiked in 1.25 mM of empty liposomes was measured and showed a linear relationship ($R^2 \geq 0.99$). Indicated is the average RFU ± SD measured on two separate days.

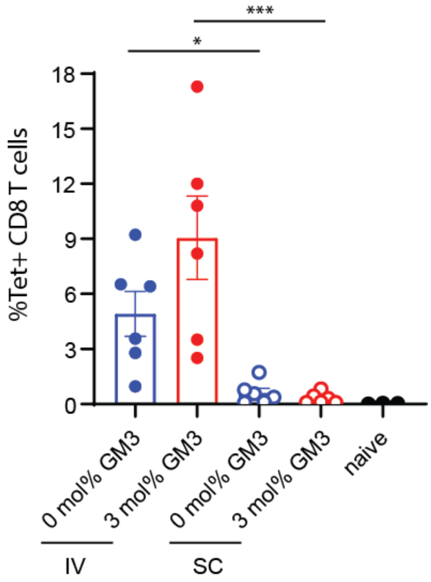

Figure S6. Intravenous administration of GM3-containing liposomes augments vaccine effectiveness. Liposomes were IV or SC administered to mice (22.5 nmol phospholipid), supplemented with adjuvant. On day 7, H-2K^b/SIINFEKL tetramer binding T cells were identified. ($n = 6$) for experimental and ($n = 3$) for the naïve group. * $p < 0.05$ and *** $p < 0.005$.

Materials and Methods:

Solid-Phase Peptide Synthesis and Characterization

Preloaded Fmoc-lys(boc)-Wang resin, 9-fluorenylmethyloxycarbonyl (Fmoc)-protected amino acids and trifluoroacetic acid (TFA) were purchased from Novabiochem GmbH (Hohenbrunn, Germany). Peptide grade dimethylformide (DMF), dichloromethane (DCM), piperidine, N,N'-diisopropylcarbodiimide (DIC), and HPLC grade acetonitrile were purchased from Biosolve BV (Valkenswaard, Netherlands). Ethyl cyanohydroxyiminoacetate (Oxyma pure) was purchased from Machester Organics ltd (Cheshire, UK). Triisopropylsilane (TIPS), BioUltra grade ammonium bicarbonate was purchased from Sigma-aldrich Chemie BV (Zwijndrecht, Netherlands). 5-Carboxyfluorescein-labelled Fmoc-lysine (Fmoc-lys(5-FAM)-OH) was purchased from APPTec (Louisville, KY, USA).

The peptides were synthesized by microwave-assisted solid phase peptide synthesis using a H12 liberty blue peptide synthesizer (CEM coporation, Matthews, NC, USA), DMF was used as the coupling and washing solvent. For coupling, Fmoc-amino acids were activated by 5eq of Oxyma pure and DIC to react with the free N-terminal amino acids in the resin for 1 minute at 90˚C. Subsequently, the Fmoc group was removed by treatment with 20% piperidine for 1 minute at 90˚C. TFA/water/TIPS (95/2.5/2.5) were used to cleave the peptide from the resin and remove the side chain protecting groups. Peptides were purified by Prep-HPLC (Waters corporation, Pleasanton, CA, USA) using Reprosil-Pur C18 column (10 µm, 250 × 22 mm) (Dr. Maisch HPLC GmbH, Germany) eluted with water-acetonitrile gradient (5%–80%) (10mM ammonium bicarbonate) in 30 minutes at flow-rate of 15.0 ml/min with UV detection at 220 nm. Purity was confirmed to be >90% by analytical ultra-high performance liquid chromatography (UPLC) (Waters Corporation, USA) using a C4 column (ACQUITYUPLC™ BEHC4 300 Å 1.7 µm, 2.1 × 50 mm) eluted with water-acetonitrile gradient (10%-80%) (10mM ammonium bicarbonate) in 7 minutes at flow rate 0.5 ml/min and UV detection at 220/280 and fluorescence excitation and detection at 280 and 340 nm, respectively. For the fluorescent peptide additional measurement was performed using excitation at 492 nm and detection at 517 nm. Mass spectrometry (MS) analysis was performed using micrOTOF-Q instrument.
